# Numerical Relations and Skill Level Constrain Co-Adaptive Behaviors of Agents in Sports Teams

**DOI:** 10.1371/journal.pone.0107112

**Published:** 2014-09-05

**Authors:** Pedro Silva, Bruno Travassos, Luís Vilar, Paulo Aguiar, Keith Davids, Duarte Araújo, Júlio Garganta

**Affiliations:** 1 CIFI²D - Centre for Research, Education, Innovation and Intervention in Sport, Faculdade de Desporto, Universidade do Porto, Porto, Portugal; 2 CIDESD, Department of Sport Sciences, Universidade da Beira Interior, Covilhã, Portugal; 3 Faculty of Physical Education and Sports, Lusófona University of Humanities and Technologies, Lisboa, Portugal; 4 CIPER, Faculdade de Motricidade Humana, Universidade de Lisboa, Cruz Quebrada Dafundo, Lisboa, Portugal; 5 Centre for Mathematics, Faculdade de Ciências, Universidade do Porto, Porto, Portugal; 6 Centre for Sports Engineering Research, Sheffield Hallam University, Sheffield, United Kingdom; 7 FiDiPro Programme, University of Jyväskylä, Jyväskylä, Finland; University of California, Merced, United States of America

## Abstract

Similar to other complex systems in nature (e.g., a hunting pack, flocks of birds), sports teams have been modeled as social neurobiological systems in which interpersonal coordination tendencies of agents underpin team swarming behaviors. Swarming is seen as the result of agent co-adaptation to ecological constraints of performance environments by collectively perceiving specific possibilities for action (affordances for self and shared affordances). A major principle of invasion team sports assumed to promote effective performance is to outnumber the opposition (creation of numerical overloads) during different performance phases (attack and defense) in spatial regions adjacent to the ball. Such performance principles are assimilated by system agents through manipulation of numerical relations between teams during training in order to create artificially asymmetrical performance contexts to simulate overloaded and underloaded situations. Here we evaluated effects of different numerical relations differentiated by agent skill level, examining emergent inter-individual, intra- and inter-team coordination. Groups of association football players (national – NLP and regional-level – RLP) participated in small-sided and conditioned games in which numerical relations between system agents were manipulated (5v5, 5v4 and 5v3). Typical grouping tendencies in sports teams (major ranges, stretch indices, distances of team centers to goals and distances between the teams' opposing line-forces in specific team sectors) were recorded by plotting positional coordinates of individual agents through continuous GPS tracking. Results showed that creation of numerical asymmetries during training constrained agents' individual dominant regions, the underloaded teams' compactness and each team's relative position on-field, as well as distances between specific team sectors. We also observed how skill level impacted individual and team coordination tendencies. Data revealed emergence of co-adaptive behaviors between interacting neurobiological social system agents in the context of sport performance. Such observations have broader implications for training design involving manipulations of numerical relations between interacting members of social collectives.

## Introduction

Collective organizational principles underlying emergence of functional behaviors have been identified in many groups of biological organisms (e.g., flocks of birds, wolf packs, ant colonies) [1], [2]. Observations of such superorganismic systems have revealed some advantages of swarming behaviors to achieve group goals, such as when feeding and maintaining member security. For instance, the labor of thousands of bees in a colony is collectively coordinated so that surrounding areas are surveyed most efficiently for food sources of nectar and pollen [3].

Human groups can also be considered as swarming superorganisms when individuals cooperate and coordinate their actions together to achieve common collective goals [4]. This sociobiological perspective can help explain various social-psychological phenomena such as the organization of labor by workers in a factory, how a traffic jam arises or the interpersonal rhythmic movements characterizing human activities like dancing or marching together.

Recently, this approach has been implemented to understand how interacting individuals coordinate their movements by detecting sensory information like the visual movement of others (see [5]–[8] as examples). Despite its obvious relevance, joint actions in the human performance domain of team sports has not received the same amount of empirical attention [9].

An *ecological dynamics* perspective to understand coordination in team sport social collectives views competing performers as biological agents functioning in integrated systems composed of many interacting subsystems (attackers and defenders) that can harness inherent self-organization tendencies to satisfy specific performance environment constraints [10], [11]. From this theoretical rationale, sports teams have been modeled as social neurobiological systems whose agents co-adapt behaviors to changing ecological constraints of performance environments to achieve competitive goals [12], [13].

These theoretical ideas have implications for current training methodologies in team sports systems seeking to promote activities of individual agents into functional coordinated performance modes needed to achieve effective competitive performance. A major principle that is paramount to achieve competitive performance goals in invasion games (like association football) is the coordinated effort to create numerical superiority in the vicinity of the ball (a type of swarming behavior) in attacking and defending sub-phases of play [14], [15]. The relevance of this universal principle for successful performance in team sports was elucidated in studies of interpersonal coordination tendencies in competing agents by Vilar et al. [16]. They analyzed tendencies to maintain offensive and defensive numerical superiority in specific spatial sub regions of the performance environment by creating sudden defensive stabilities and offensive opportunities through collective behaviors during competitive football matches. More successful performance outcomes during attacking and defending sub-phases of play were directly related to the relative number of opposing players adopting a spatial location nearer to the ball (see [16]).

The creation of local numerical superiority through collective swarming behaviors when teams possess equal numbers of players is difficult to achieve without effective team coordination developed during training. To heighten awareness of such emergent interpersonal coordination tendencies during performance, coaches seek to simulate numerical asymmetries by designing training programs in which an attacking team is overloaded and a defending team is underloaded, respectively, containing more and fewer players. In such training practices, players learn to explore the interpersonal interactions that shape how different numerical relations can be suddenly created during attacking and defending sub-phases of play. In team sports training programs, small-sided and conditioned games (SSCGs) provide an important task vehicle used to constrain the emergence of interpersonal tactical behaviors in system agents through manipulations of numerical relations (in this paper such practice tasks are designated as small-sided “*and conditioned*” games because other constraints, besides field dimension, can be manipulated in order to shape specific behaviors; e.g., player numbers, rules, etc.).

The theoretical reasoning behind use of SSCGs is that they are simulations created during training to help team sports players to learn how to satisfy different constraints on their emergent collective behaviors. Indeed, they are important vehicles to aid sports teams, as complex neurobiological systems, to exploit inherent tendencies for co-adaptation and self-organization [1], [17]. These properties of complexity have also been used to explain how agents in social neurobiological systems evolve and adapt their behaviors to satisfy evolutionary constraints [12]. Like such systems, players in sports teams use functional, context-dependent information to regulate their collective behaviors in dynamic performance environments. Previous research suggests that these emergent interpersonal coordination tendencies are predicated on perception of the action possibilities of nearby players [13], [18] and the ball's displacement over space and time [19], which *afford* specific actions [20].

The concept of a*ffordances*, as originally postulated by Gibson [21], refers to the possibilities for action that emerge from the interactions of an organism with its environment. This concept is useful for explaining emergence of interpersonal interactions in team sports since the ability to perceive action possibilities for self in humans is complemented by their capacity to perceive another individual's *affordances* [22] and intentions [23]. As Gibson argued, the richest affordances are provided by interactions with others, since ‘behavior affords behavior’ (p135), signifying how the environment itself can also be perceived in relation to self and another person's abilities (see Witt et al. [24], [25]). Accordingly, an *ecological dynamics* approach advocates that the interpersonal synergies [26] established between system agents in sports teams can emerge through the perception of *shared affordances*. These are possibilities for action that players collectively perceive through their interacting behaviors with their colleagues and adversaries and that can be effectively designed into SSCGs [27], [28]. For instance, a ball carrier in a SSCG can perceive a possibility for passing the ball to an unmarked teammate by also perceiving for him/her a possibility to receive the ball. In this example, both coordinating players perceive *affordances* for one another. The coordinated action of passing and receiving a ball composes a *shared affordance* that is specified to individuals forming a single synergy in different ways (see [17] for a detailed explanation on how shared affordances and synergies may form the basis of coordination of team tactical behaviors). Affordances are dynamic and coordination tendencies between interacting teammates, and between them and their opponents, can change due to the creation of overloads in attack and defense through swarming. In this sense, learning to suddenly outnumber the opposition by overloading in specific spatial areas of a SSCG training environment can help players to create numerical advantages by selectively picking up *shared affordances* that satisfy a team's momentary goals. This interactive process supports the emergence of swarming behaviors and the team's complex tactical performance [28]. However, the tactical behaviors exhibited by agents in team sports, like association football, that emerge at the group level (i.e., when swarming), due to the creation of unbalanced numerical relations, have seldom been investigated. In one exception, Travassos et al. [19] observed how, in the team sport of futsal, agents in a defending underloaded team, in a 5v4 + goalkeeper (gk) game context (a common situation where the goalkeeper of an attacking team plays as an outfield player to create a numerical advantage over a defending team), swarmed around the ball and protected their goal space. The players achieved this performance goal by synchronizing their own movements with the ball's lateral displacements in front of their own goal area. More information about team behaviors during asymmetrical game contexts is clearly needed. Such contexts emerge quite often in many team invasion games besides association football and futsal, such as waterpolo, rugby union, handball or hockey, when one player is ‘sin-binned’ or sent off temporarily or definitely. This information may be an asset for designers of team training programs to enhance understanding of emergent collective tactical behaviors, skill acquisition and decision-making as a consequence of the interplay of specific ecological constraints in a learning environment [29].

An important, related question concerns whether tactical behaviors emerging from manipulations of numerical relations between competing system agents might be influenced by performer skill level. Silva et al. [30] demonstrated that players of distinct competitive levels displayed different spatial distributions and movement oscillations on field during SSCGs, as a consequence of their skill level. Hence, it is important to understand how players' skill levels, within the ecological constraints of specific performance environments, can influence the interpersonal coordination tendencies afforded by differing numerical relations during training in SSCGs.

Previous investigations of coordination tendencies in team sport systems have revealed a number of reliable individual and compound performance variables [1] for assessing interpersonal coordination tendencies at different levels of system analysis (e.g. inter-individual, intra- and inter-team coordination levels).

Inter-individual coordination analyses have focused on cooperative tendencies amongst team members, providing information on the division of labor between agents [31]. For instance, recent work examined players' dominant regions in team sports of football and futsal by quantifying each individual's major ranges [32] and Voronoi cells [33], which depict the division of labor amongst team members. At a more macroscopic level of analysis, researchers have measured, for instance, teams' geometrical centers (or centroids) and stretch indices to assess intra-team coordination patterns [34], [35]. These variables provide complementary information about emergent agent coordination tendencies, with the centroid highlighting the relative position of one team on field and the stretch index plotting the dispersion of players around the team center. *Intra-team analyses* also support identification of different characteristics of the coordination tendencies within each of the opposing teams.

At a larger scale of analysis, inter-team coordination focuses on the coordination patterns emerging *between* opposing teams and highlights the competitive interactions between players and teams to achieve specific performance goals. Knowledge about inter-team coordination has been obtained, for example, by quantifying distances between competing teams' centroids during performance [36]. An alternative to studying inter-team coordination tendencies involves analysis of specific sub-grouping alignments in sports teams, such as line-forces [37], [38]. Line-forces provide (geometrically) an estimate of team cohesion (e.g., between players in a defensive line), representing the functional form in which players' attacking and defensive actions are co-aligned and co-organized according to specific team sectors (longitudinally, in attacking, midfield and defending team sectors or laterally, on the wings and middle axis of the field) [39]. Through this conception, inter-team coordination tendencies can be studied according to specific sub-grouping alignments, by measuring the distance between competing lines of players on field.

To summarize, due to the lack of empirical work addressing effects of different numerical relations on coordination tendencies in systems of interacting team sports players, we sought to analyze tactical behaviors emerging from swarming tendencies in SSCGs. To achieve this aim, we investigated whether different numerical relations between competing teams in association football impacted inter-individual, intra- and inter-team tactical behaviors (i.e., emerging in overloaded and underloaded teams), as well as the relative influence of player skill level, specifically on the: (i) players' division of labor, (ii) teams' relative positioning on field; (iii) teams' dispersion; and (iv), inter-team distances at specific locations.

## Methods

### Participants

Twenty male, youth football players (under-19 yrs) participated in this study, divided into two groups according to skill level (NLP, national-level of performance or RLP - regional-level of performance). Ten participants in the NLP group (mean age: 17.64±0.67 yrs) played at a national-level in a top club from their country of origin (playing experience: 9.55±0.52 yrs). Two participants from this group played for their country's under-19 yrs national team. Participants in the RLP (age: 17.91±0.3 yrs) competed at a regional-level competition (playing experience: 9±1.9 yrs). All players or legal guardians (when under age) provided written informed consent to participate in the experiment. All procedures followed the guidelines stated in the Declaration of Helsinki and were approved by the Ethics Committee of the Faculty of Sports of Porto University.

### Data collection

Within each group, participants were assigned to one of two technically-equivalent teams of five players by their coaches. Participants performed in three SSCGs in which the numerical relations of the two teams were manipulated. The first SSCG consisted of a 5-a-side plus goalkeeper (Gk) game (5v5+Gk). The goalkeepers played inside an area marked 5 m from the goal line and extending across the pitch width, while defending a 6×2 m (height × width) football goal. The team without a goalkeeper defended three mini-goals (1.2×0.8 m) located on the end line of the pitch. In the second and third SSCGs, the team playing with a goalkeeper was reduced to four (5v4+Gk) and three (5v3+Gk) outfield players, respectively. In accordance with pedagogical principles, this team was denominated the *underloaded* team, while the team with the same number of players was denominated the *overloaded* team. The objective of teams in all SSCGs was to score as many goals as possible and to prevent the opposing team from scoring goals (regardless of numerical overloaded or underloaded performance conditions and of the momentary score in the SSCG). The underloaded team attacked mini-goals without goalkeepers to maintain their chances of scoring when playing with a numerical disadvantage. Table 1 shows that both groups performed a similar number of shots and goals in all treatments. The effectiveness of the shots of both skill groups' underloaded teams confirms that, even when playing under a disadvantage of two fewer players, the chances of scoring were high (given that there was no GK). Effectively, there existed an attacking risk to the overloaded teams when possession was lost.

**Table 1:** Number of shots and goals obtained by teams on each of the small-sided and conditioned games (SSCGs), according to skill.

SSCGs	National-level players				Regional-level players			
	Overloaded team		Underloaded team		Overloaded team		Underloaded team	
	Shots	Goals	Shots	Goals	Shots	Goals	Shots	Goals
**5v5**	6	1	4	4	3	2	4	4
**5v4**	8	2	2	2	8	3	2	2
**5v3**	11	5	4	4	12	5	5	5

The teams attacking the goal with a goalkeeper (overloaded teams) performed more shots in all treatments, with the exception of the regional-level players in the 5v5 treatment. The teams defending mini-goals (overloaded teams) always lost when playing under balanced numerical relations. This reflects the larger risks taken by this team defending goals without a goalkeeper. The underloaded teams performed fewer shots but the opportunities to shoot always ended in goals, depending only on the skill of the player to hit the target after a shooting opportunity was created.

The length and width dimensions of the playing area were reduced, relative to official football field dimensions, to 47.3×30.6 m, given the number of players involved in the SSCG [40]. Each match lasted for 6 minutes interspersed with 6 minutes periods of rest to minimize the influence of fatigue on participants. During recovery periods, participants were allowed to recover actively at will and rehydrate. Time-motion analysis obtained through continuous GPS tracking showed similar physical profiles across treatments for the players of the overloaded teams. The players of the underloaded teams tended to augment the total distance covered and high intensity running activities when playing under a numerical disadvantage of two players (5v3 + Gk). These data signaled that accumulated fatigue of participants did not bias the results of the experiment.

Coaches were instructed not to provide any sort of encouragement or feedback to the players, before and during periods of data collection, as it could have distorted levels of practice intensity in individual participants [41].

All outfield players carried an unobtrusive global positioning tracking device (SPI Pro, GPSports, Canberra, Australia) to record their movement displacements with individual positional data (2D) at a sampling frequency rate of 15 Hz. The reliability of such devices has been previously well documented [42], [43].

The performance area used in all treatments was calibrated with the coordinates of four GPS devices stationed in each corner of the pitch for approximately 2 minutes. The absolute coordinates of each corner were calculated as the median of the recorded time series, providing measurements that were robust to the typical fluctuations of the GPS signals. These absolute positions were used to set the Cartesian coordinate systems for each pitch, with the origin placed at the pitch center. Longitudinal and latitudinal (spherical) coordinates were converted to Euclidean (planar) coordinates using the Haversine formula [44]. Fluctuations in player positioning measures were reduced using a moving average filter with a time scale of 0.2 seconds and data resampling was employed to synchronize the time series of all players within each game.

### Data analysis

Inter-individual coordination tendencies were analyzed measuring the dominant region of each player on field. To this effect, the major ranges of each player's displacement were calculated as the ellipse centered at the 2D mean location of each player (i.e., the locus), with semi-axes being the standard deviations in the longitudinal and lateral directions for each entire SSCG [32]. Analysis of ellipse shapes provides qualitative evaluation of the main directions of players' movements, their distribution and relative positioning on field. The areas of the ellipses were also calculated to furnish quantitative information of the amount of space that was under the dominant region of each player.

To establish intra-team coordination we calculated the stretch index (SI) and the centroid's distance to the goal center (CdtG – in the case of the underloaded team) and to the end line where the mini-goals were placed (CdtMG – in the case of the overloaded team). The SI was calculated as the mean value of the distances of each player to their team's centroid, whereas the centroid was calculated as the average position of all outfield players of one team.

Inter-team coordination was examined through analysis of the distances separating the teams' horizontal and vertical opposing line-forces. We opted to record this variable instead of measurements of centroid distance values because the former does not capture the existence of eventual differences in the players' interactive behaviors at specific team locations (e.g., wings and sectors).

Each team's horizontal lines were calculated by averaging the longitudinal coordinate values of the two players furthest from, and nearest to their own goal line, which corresponded to the forward and back lines, respectively. Similarly, the vertical line-forces of each team were computed by averaging the mean lateral coordinates of the players furthest to the left and to the right on the pitch, corresponding to the left and right lines, respectively. Due to the small number of players participating in the SSCGs, only the wing lines and attacking and defending sectors were analyzed. Hence, the time series of the distances between forward-back and left-right lines of opposing teams were calculated according to team sectors, as follows: (i) dtH1 - distance between the back line of the underloaded team and the forward line of the overloaded team; (ii) dtH2 - distance between the forward line of the underloaded team and the back line of the overloaded team; (iii) dtV1 - distance between the left line of the overloaded team and the right line of the underloaded team; and (iv), dtV2 - distance between the right line of the overloaded team and the left line of the underloaded team.

**Figure 1:**
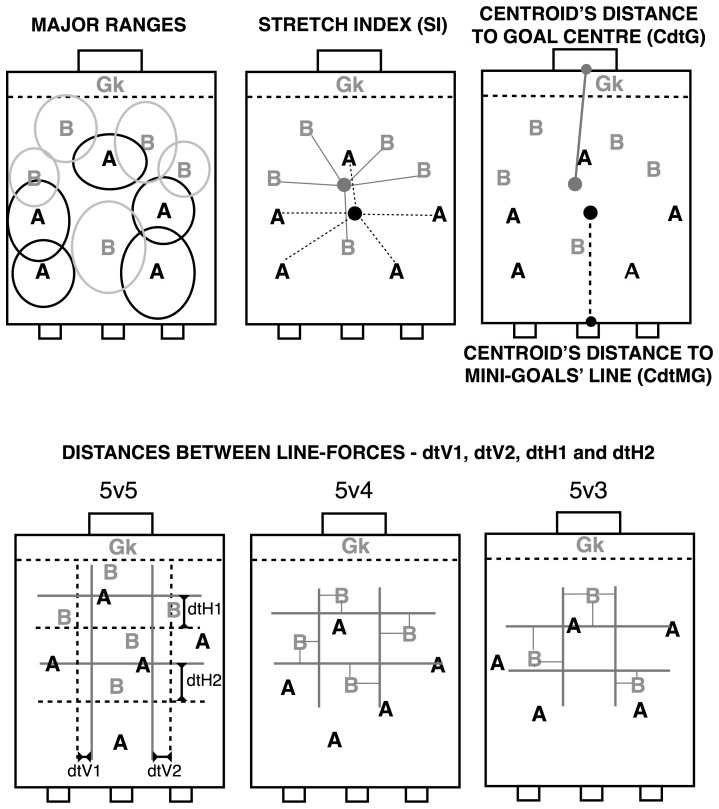
Illustration of inter-individual (major ranges), intra- (stretch index and centroids' distance to goals) and inter-team (distances between line-forces) variables used. The lower left panel illustrates the distances between opposing horizontal (dtH1 and dtH2) and vertical line-forces (dtV1 and dtV2) in the 5v5 condition. The lower middle and right panels illustrate the calculation of horizontal and vertical line-forces in the underloaded team with 4 and 3 players, respectively. All fields used were 47.3×30.6 m (length × width). The overloaded team attacked the goal with a goalkeeper and the underloaded team attacked the mini-goals.

All variables used in this study capture the interpersonal coordination tendencies at different levels of system analysis (inter-individual, intra- and inter-team coordination levels), as illustrated in Figure 1.

### Statistical procedures

Mean ± standard deviations values of the ellipse areas were calculated for the numerically overloaded and underloaded teams according to skill level and different numerical relations. Given that the performance data of each team were analyzed separately and that there was a small number of participants per team (5 players in the overloaded team and 5, 4 and 3 players in the underloaded team), no inferential statistics were used to analyze major ranges areas.

The time series values of intra- (SI, CdtMG and CdtG) and inter-team (dtH1, dtH2, dtV1 and dtV2) variables were compared statistically through analysis of variance methods. Given that these time series are of a stochastic nature, the successive measures were only partly determined by previous values. Hence, the assumption of independence of the observations required to run the ANOVAs was overcome by sampling uncorrelated data points. To this effect, for each variable, time series values interspersed with a time interval (*t*) were selected to ensure that each observation recorded would be uncorrelated with previous selected values. Thus, the time series were fitted to autoregressive models (AR) of various orders (1 to 10) with the argument that, if the data came from an AR(*p*) process, then *t≥p.* Thus, *t* can be estimated by solving the first *p* Yule-Walker equations with correlations estimated using the sample autocorrelation coefficients. The order of the process is, then, estimated to minimize the Schwarz's Bayesian criterion that represents a trade-off between the fit of the AR model and the number of parameters estimated [45]. Through the AR processes, 82% of the estimates were equal to *p* = 4 and lower. Based on this finding, measures of all intra- and inter-team variables were sampled at every 4 seconds of play (totaling 90 independent measures per variable). After this procedure, for each SSCG and group, we obtained a set of independent game situations containing identical variances, means and standard deviations of the original variables' time series.

Two-way ANOVAs were then conducted to examine the effect of skill (2 levels: NLP and RLP) and numerical relations (3 levels: 5v5, 5v4 and 5v3) on each intra- and inter-team variable. Statistical analysis of intra-team variables was run separately, according to the teams' numerical advantage – overloaded and underloaded teams. Given the large sample of data points analyzed (540 data points per variable), all statistical comparisons reported outcomes below the conventional statistical significance alpha value of *P* = 0.05. Thus, we focused on the magnitude of the effects (here reported as partial eta squared - *η^2^*) obtained with the ANOVAs, following Cohen's guidelines [46]: (i) 0.01≤*η^2^*<0.06 – small effect; (ii) 0.06≤*η^2^*<0.14 – moderate effect; and (iii) *η^2^*≥0.14 – large effect. Bonferroni post-hoc pairwise comparisons and interaction effects were implemented when moderate or large effects of *η^2^* were identified.

For simplicity, when playing with equal number of players, the team attacking the goal with a goalkeeper and the team attacking the mini-goals were also denominated as overloaded and underloaded teams, respectively.

## Results

### Inter-individual coordination (major ranges)

**Figure 2:**
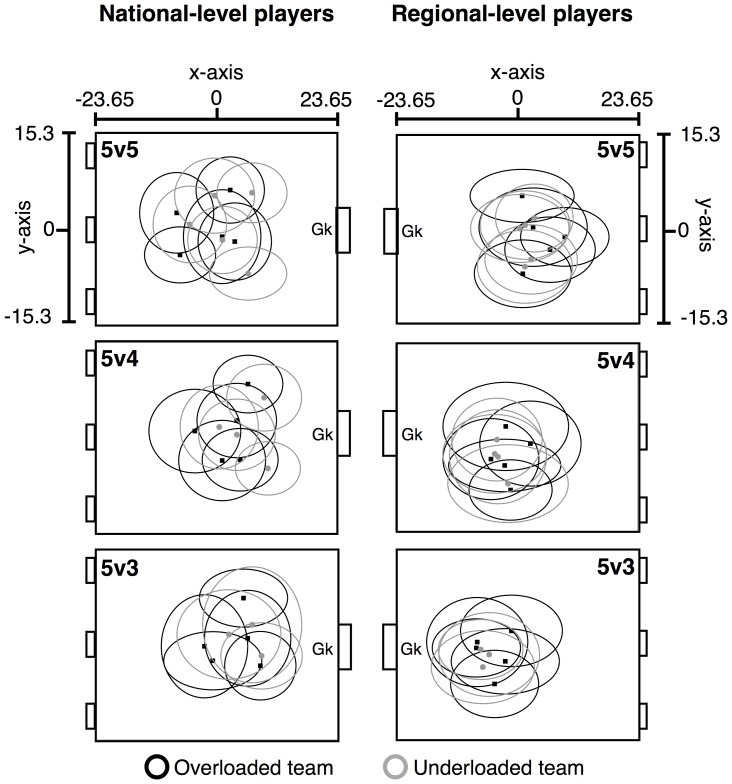
Major ranges of national- (NLP) and regional-level players (RLP) in each SSCG. Black and grey ellipses depict the major ranges of the overloaded and underloaded teams, respectively. Overloaded teams attack the goal defended by a goalkeeper. Lateral (y-axis) and longitudinal (x-axis) depict field coordinates.

Figure 2 illustrates the major ranges and means ± standard deviations of the ellipse areas in each treatment for each group of players.

**Figure 3:**
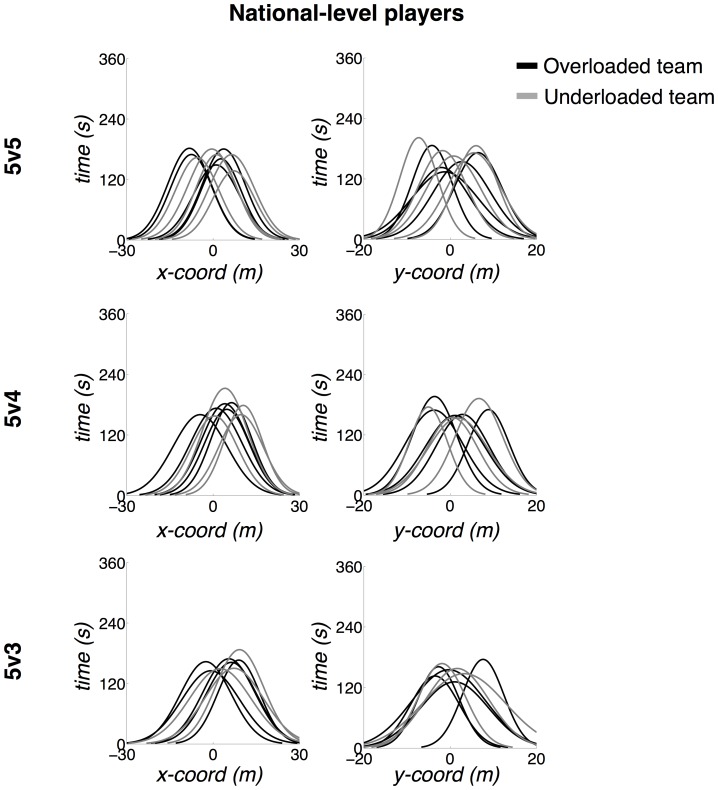
Normal density function of the players' distribution along the x- (longitudinal) and y-axes (lateral) – national-level players (NLP) distribution. Field coordinates vary between −23.65–23.65 and −15.3–15.3 for the x- and y-axes, respectively. The origin (0, 0) corresponds to the field center.

**Figure 4:**
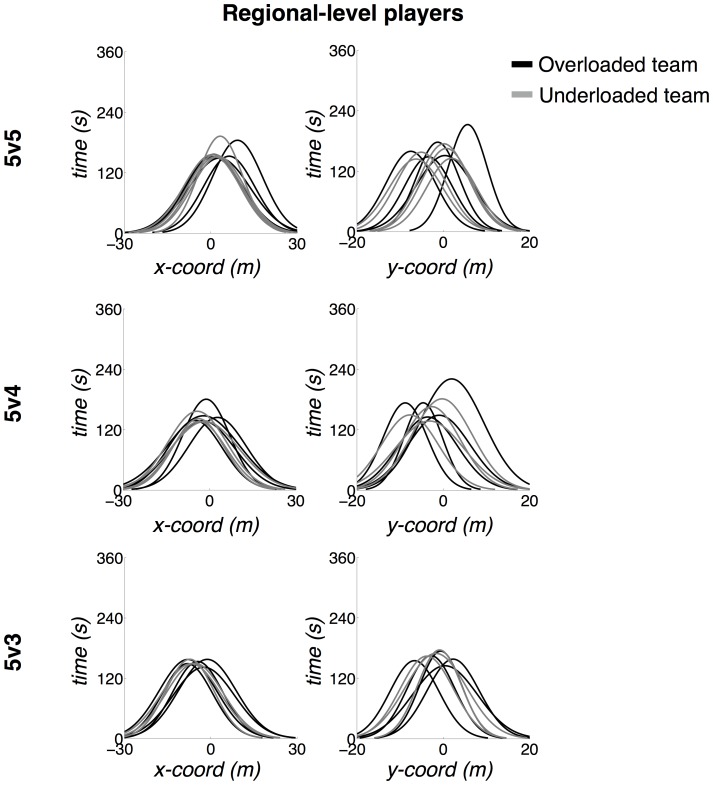
Normal density function of the players' distribution along the x- (longitudinal) and y-axes (lateral) – regional-level players (RLP) distribution. Field coordinates vary between −23.65–23.65 and −15.3–15.3 for the x- and y-axes, respectively. The origin (0, 0) corresponds to the field center.

The ellipse areas of the RLP were more superimposed, displaying different shapes, compared to the NLP skill level. The latter displayed ellipses that were more rationally distributed according to corridors (left and right) and sectors (forward and back), presenting rounded shapes. Analysis of the distribution of players' movement coordinates in the x- (longitudinal) and y-axis (lateral) in Figures 3 and 4 confirm that NLP displayed differentiated distributions in the x-axis (Figure 3). In contrast, the RLP tended to play on very similar longitudinal coordinates of the field, only varying their positioning along the y-axis (Figure 4). The RLP also presented broader distributions along the x-axis, which caused their ellipses to be oval-shaped.

**Figure 5:**
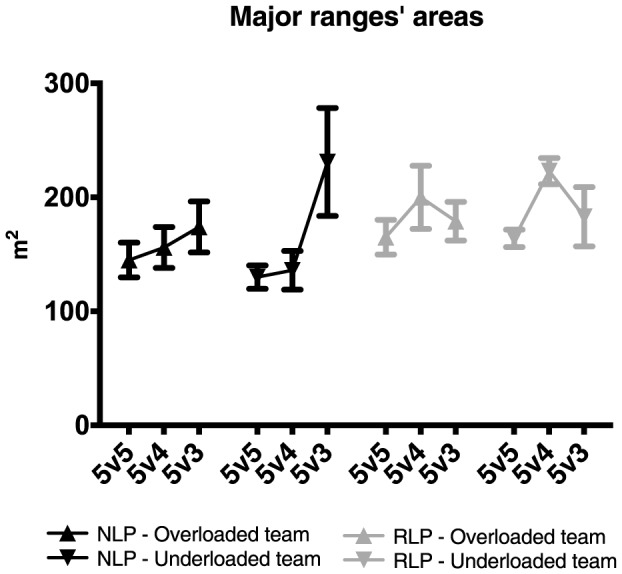
Major ranges areas of national- (NLP) and regional-level players (RLP) in each treatment. Error bars represent the standard error of the mean.

Additionally, the mean areas of ellipses in the NLP group presented a similar trend for both the overloaded and underloaded teams in the 5v5 and 5v4 condition (see Figure 5). In the 5v3 condition, both teams increased their mean areas but with the underloaded team displaying much larger mean ellipse areas than the overloaded team. This was evident in the ellipses of two out of the three players and also in the distribution of their coordinates in the y-axis (Figure 3) that was broader in the 5v3 treatment.

The RLP group displayed different mean areas across treatments for both teams, but identical mean areas for both underloaded and overloaded teams. The larger mean areas were registered in the 5v4 treatment.

### Intra-team coordination (stretch index and centroids' distance to own goal and mini-goals' line)

**Figure 6:**
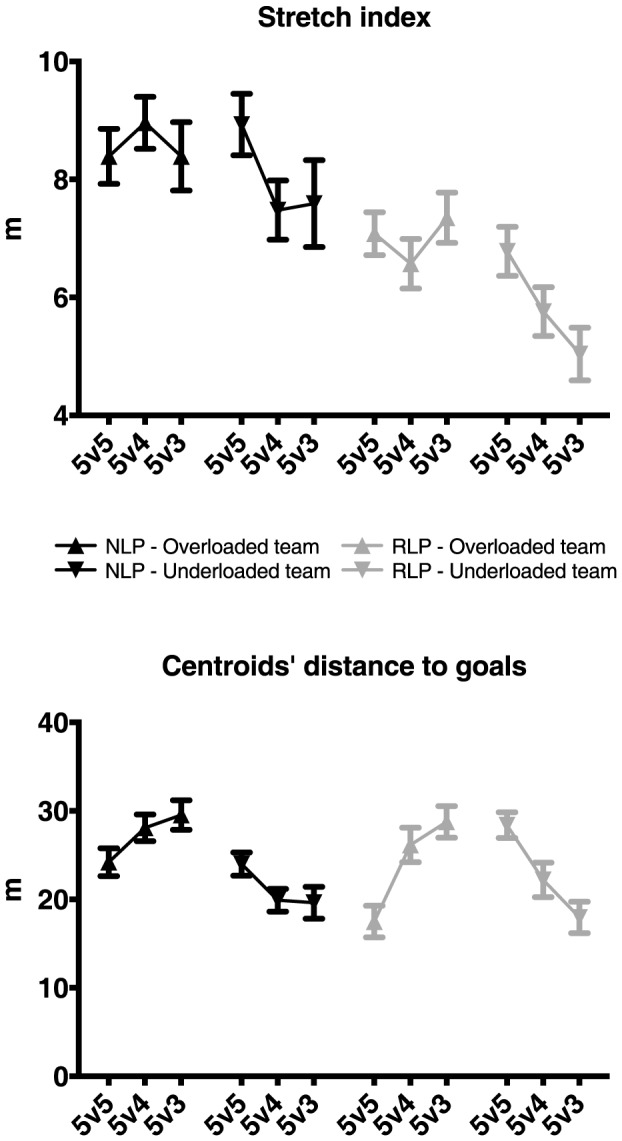
Mean stretch index (SI) and centroids' distance to goals' center (CdtG) and mini-goals line (CdtMG) of overloaded and underloaded teams across treatments and expertise groups. Error bars represent the standard error of the mean.

Statistical analysis of SI showed a moderate effect of skill for the overloaded team, *F*(1,534) = 71.759, *P*<0.001, *η^2^* = 0.12. Higher mean values of SI were found for NLP (*M* = 8.58, *SE* = 0.19) than RLP (*M* = 7, *SE* = 0.19, see Figure 6, upper panel). No significant effects were observed for numerical relations, or the interactions (Table 2).

**Table 2:** ANOVA effect sizes.

Variables	Skill level (SL)	Numerical relation (NR)	SL × NR
SI	Overloaded: *η^2^* = 0.12*; Underloaded: *η^2^* = 0.16**	Overloaded: *η^2^* = 0.001; Underloaded: *η^2^* = 0.07*	Overloaded: *η^2^* = 0.02; Underloaded: *η^2^* = 0.005
**CdtMG**	*η^2^* = 0.04	*η^2^* = 0.16**	*η^2^* = 0.02
**CdtG**	*η^2^* = 0.01	*η^2^* = 0.14**	*η^2^* = 0.02
**dtH1**	*η^2^* = 0.001	*η^2^* = 0.02	*η^2^* = 0.05
**dtH2**	*η^2^* = 0.01	*η^2^* = 0.07*	*η^2^* = 0.02
**dtV1**	*η^2^* = 0.004	*η^2^* = 0.09*	*η^2^*<0.001
**dtV2**	*η^2^* = 0.06*	*η^2^* = 0.01	*η^2^* = 0.002

Main effects of skill level and numerical relation and interaction effects of skill level x numerical relation on: (1) stretch index (SI); (2) centroid's distance to mini goals line (CdtMG); (3) centroid's distance to goal (CdtG); (4) horizontal lines' distances (dtH1 and dtH2) and (5) vertical lines' distances (dtV1 and dtV2).

*Moderate effect.

**Large effect.

ANOVA of SI for the underloaded team presented a large effect of skill level, *F*(1,534) = 101.23, *P*<0.001, *η^2^* = 0.16, revealing higher mean values of SI for NLP (*M* = 7.99, *SE* = 0.21) than RLP (*M* = 5.86, *SE* = 0.21). Analysis of SI in the underloaded team also revealed a moderate effect of numerical relations, *F*(2,534) = 19.66, *P*<0.001, *η^2^* = 0.07. Bonferroni post hoc analyses showed higher mean values of SI for 5v5 (*M* = 7.85, *SE* = 0.26), than for 5v4 (*M* = 6.62, *SE* = 0.26, *P*<0.001) and 5v3 (*M* = 6.31, *SE* = 0.26, *P*<0.001). Interaction effects were negligible (see Table 2).

Concerning CdtMG, ANOVA revealed a large effect of numerical relations, *F*(2,534) = 50.26, *P*<0.001, *η^2^* = 0.16. Lower CdtMG mean values were found for 5v5 treatments (*M* = 20.85, *SE* = 0.86) than for 5v4 (*M* = 27.13, *SE* = 0.86, *P*<0.001) and 5v3 (*M* = 29.15, *SE* = 0.86, *P*<0.001, see Figure 6, lower panel). Skill level and interaction effects were not statistically significant (Table 2).

Concerning the CdtG of the underloaded team, a large effect was also obtained for numerical relations, *F*(2,534) = 43.65, *P*<0.001, *η^2^* = 0.14, with larger mean values displayed for 5v5 (*M* = 26.19, *SE* = 0.81) than for 5v4 (*M* = 21.05, *SE* = 0.81, *P*<0.001) and 5v3 (*M* = 18.78, *SE* = 0.81, *P*<0.001). Differences between 5v4 and 5v3 treatments were also observed (*P* = 0.01, *SE* = 0.81, see Figure 6, lower panel). No significant effects of skill level and interactions were observed.

### Inter-team coordination (distances between opposing vertical and horizontal line-forces)

**Figure 7:**
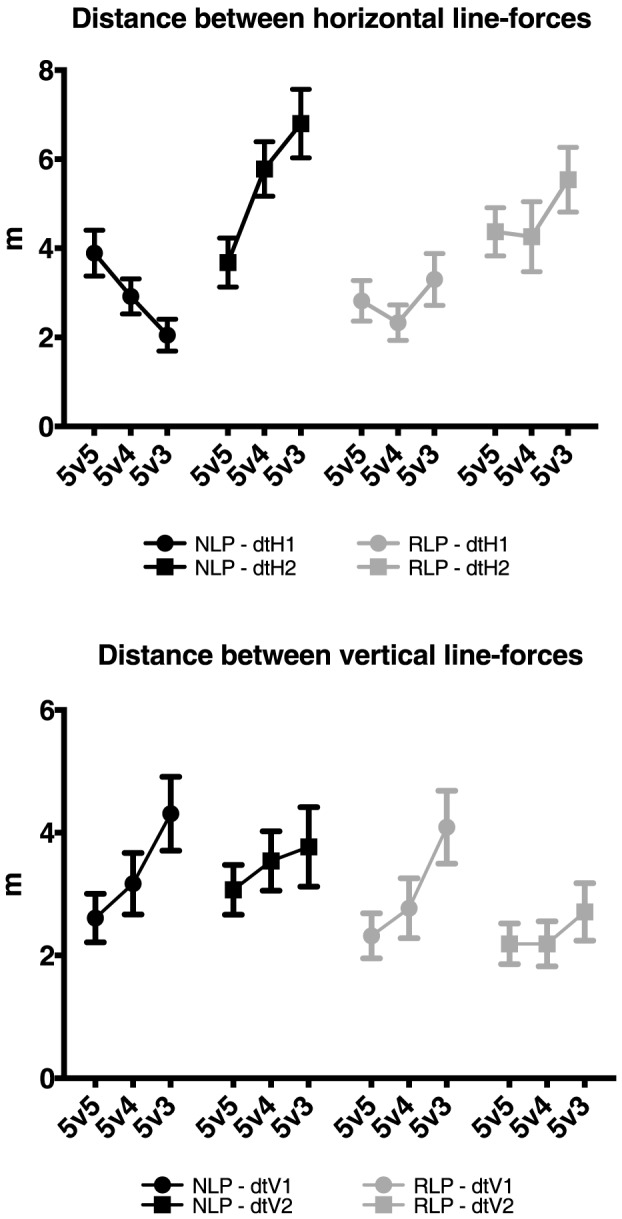
Mean distances between horizontal (upper panels – dtH1 and dtH2) and vertical lines (lower panels – dtV1 and dtV2) across treatments and expertise groups. Error bars represent the standard error of the mean.

Despite not being sufficiently large to be conventionally considered a significant statistical effect, it is worth noting the interaction effect emerging for skill level and numerical relations, for confrontation of horizontal lines dtH1, *F*(2,534) = 14.17, *P*<0.001, *η^2^* = 0.05, since it may have some practical significance (see discussion). Post-hoc analysis showed higher mean values of dtH1 for NLP (*M* = 3.89, *SE* = 0.32) than for RLP (*M* = 2.82, *SE* = 0.32, *P*<0.001) in the 5v5 treatment (Figure 7, upper panel). In contrast, in the 5v3, post hoc analysis reported lower mean values of dtH1 for NLP (*M* = 2.05, *SE* = 0.32) than RLP (*M* = 3.3, *SE* = 0.32, *P*<0.001). No differences were found between groups in the 5v4 treatment (*P* = 0.07).

Concerning dtH2, a moderate effect of numerical relation was observed, independent of skill level, *F*(2,534) = 20.19, *P*<0.001, *η^2^* = 0.07. Post-hoc analysis showed lower mean values of dtH2 in the 5v5 (*M* = 4.02, *SE* = 0.34) than in the 5v4 (*M* = 5.02, *SE* = 0.39, *P* = 0.01), and in the 5v3 (*M* = 6.17, *SE* = 0.34, *P*<0.001). Significant differences were also found between the 5v4 and 5v3 treatments (*P* = 0.002).

Analysis of variance of dtV1 also registered a moderate effect for numerical relations, independent of skill level, *F*(2,534) = 25.22, *P*<0.001, *η^2^* = 0.09, but small effects for skill level and interactions (Table 2). Post-hoc tests revealed higher mean values of dtV1 for 5v3 (*M* = 4.2, *SE* = 0.25) than for 5v4 (*M* = 2.97, *SE* = 0.25, *P*<0.001) and 5v5 (*M* = 2.47, *SE* = 0.25, *P*<0.001, see Figure 7, lower panel).

The ANOVA of dtV2 revealed a moderate effect for skill level, *F*(1,534) = 33.4, *P*<0.001, *η^2^* = 0.06, with larger mean values observed in the NLP (*M* = 3.46, *SE* = 0.19), compared to the RLP group (*M* = 2.36, *SE* = 0.19).

## Discussion

In this study we adopted an ecological dynamics perspective to investigate how players in football teams, here viewed as multi-agent neurobiological systems, co-adapted performance behaviors (by swarming) to the imposition of distinct numerical relations during SSCGs. Values of inter-individual (major ranges), intra- (stretch index and centroids' distance to goals) and inter-team (distance between horizontal and vertical line-forces) coordination patterns were analyzed during performance in SSCGs played under different numerical relations (5v5, 5v4 and 5v3). Additionally, differences in skill level were observed, between national- and regional-level players, to understand how skill level interacted with numerical relational constraints.

### Inter-individual coordination within the team

The lower superimposition of the NLP ellipses (see Figures 2 and 3) reflected a more balanced occupation of different sections of the field, a basic principle required for successful performance in invasion team sports [15]. This process was not as clear in the RLP group whose major range tendencies reflected a poorer division of labor according to specific zones of the field [1].

Analysis of ellipse shapes also supported the assumption that the NLP participants were more tactically balanced. Their rounded shapes reflected the similarity of movement amplitudes in the longitudinal and lateral direction. In contrast, RLP participants tended to present larger movement amplitudes in the longitudinal direction, as specified by the oval-shaped ellipses displayed and also by the distribution of their movements in the longitudinal axis in all treatments (see Figure 4). Considering the fact that, in general, they also possessed wider major ranges areas, it seems that these players performed more runs in the longitudinal direction than in the lateral plane. Clearly, skill level constrained the perception of different action possibilities for each group and the distinct interaction possibilities captured by the division of positional roles on field (i.e., distinct shared affordances).

In the 5v3 treatment the NLP underloaded team presented a considerable larger mean area than the overloaded team. In fact, this was the largest mean ellipse area of all treatments and groups. This outcome can be interpreted as the capacity of NLP participants to cover a wider playing area when playing under a strong numerical disadvantage (with two players less than the opposition). Indeed, when playing against a numerical disadvantage of only one player (5v4), the areas covered were similar to when they played in a numerical balance. This finding implies that for the NLP group, a numerical difference of only one player was not enough to change the players' interpersonal coordination tendencies, probably due to their superior capacity of working together to compensate for the missing player. The larger mean ellipse areas observed in the NLP overloaded team during the 5v3 treatment might be related to the fact that more free space was available to be exploited since the opposing team had two fewer players.

Concerning the RLP group, it is not clear why these players displayed larger mean ellipses areas in the 5v4 and not in the 5v3 treatments and more research is needed to clarify understanding of this effect.

In general, results on inter-individual coordination tendencies suggested that skill level was determinant in the perception of different possibilities for action and in constraining decisions [30] emerging from each group of participants regarding their division of labor on field, here represented by distinct movement patterns and territorial occupation.

### Intra-team coordination

The NLP group presented larger dispersion values than the RLP group in all treatments and teams (overloaded and underloaded team) and thus, a tendency to play in a more stretched way. However, playing against one or two fewer players did not provoke any significant changes in the dispersion values of both groups' overloaded teams.

The underloaded team of the NLP played in a more stretched way than the RLP team, perhaps reflecting their ability to spread out and cover the width of the field, when playing with a numerical disadvantage. In fact, during the defensive phase it is important that the defending team contracts to close down space near the ball but covering, at the same time, potential openings for the oppositions' passing lines. This observation signifies that players need to be spread out enough to be adjacent to different ball trajectories, according to the attackers' positions. Our findings concurred with data reported by Travassos et al. [47]. They showed that in non-intercepted passes in futsal, the distance of the second defender to the ball carrier decreased while the distance to the ball trajectory increased. In this sense, players can defend more efficiently if they are able to cover and press most of the possible passing lines available for an opponent, which implies that they should not be excessively contracted as a coordinating unit. By continually readjusting their positioning, based on defensive action possibilities for themselves and for teammates (e.g., covering space, closing passing lines, marking opponents, etc.), players can work to maintain the symmetry between competing teams in underloaded game contexts.

Nonetheless, in the underloaded teams, the values of SI decreased significantly across treatments, independent of skill level, signifying that the teams of both groups tended to contract when they were reduced in number. This finding was likely due to the fact that they spent more time performing defensive actions when underloaded. The NLP, however, presented similar dispersion values when playing with one and two players fewer, while the RLP group teams always tended to contract every time one player was omitted. Following the previous results of inter-individual coordination it seems plausible that this team maintained similar dispersion values through enlargement of their players' dominant regions. Thus, the same collective behavioral patterns were secured by distinct interpersonal coordination modes. This is another important characteristic of biological systems – the ability to degenerate behavior to satisfy different ecological constraints [48].

Concerning the teams' relative positioning on field (depicted by the centroid distance to the goal line), results showed that, as the numerical difference between teams increased, the overloaded team players moved further away from their mini-goals and approached the underloaded team's goal. This behavior was evident for both groups. In contrast, underloaded team players tended to be attracted backwards on field to defend their goal. Thus, the overloaded teams managed to acquire space near the opposing team's goal and forced them to move backwards. This result is in accordance with data reported in the study of Travassos et al. [49] in 5v4+Gk performance contexts in futsal. This coordination tendency was evident when underloaded teams were reduced by one player, while presenting similar CdtMG and CdtG values in the 5v4 and 5v3 treatments. These findings indicated the conditions that facilitated multi-agent collectives to act in a collectively coordinated manner, once players had become attuned to information from their opponents' actions.

Synthesizing these main findings, the manipulation of numerical asymmetries on field during training tasks influenced the dispersion values of the teams being underloaded and the relative positioning of both teams on field. Overloaded teams tended to advance on field when opponents lost players, but without impacting in their dispersion values. In contrast, underloaded teams tended to retreat nearer to their own goal and contract in order to protect it. In both situations, the data suggested that players were collectively attuned to shared affordances. Playing with fewer players might have offered fewer opportunities to keep possession of the ball and possibly constrained the emergence of a more compact defensive block. In contrast, playing with more players probably offered more possibilities for maintaining possession and attacking the opposition goal (which is typically the main performance behavior intended to be promoted through the creation of overloads). Skill level seemed most influential on the way each group of players spread out on field during play, although both groups presented similar trends in their emergent behaviors.

### Inter-team coordination

With regards to the distances between horizontal line-forces, results showed that the NLP participants reduced the distance value dtH1 (i.e., distance between the back line of the underloaded team and the forward line of the overloaded team) as the numerical difference between both teams increased. The opposite effect emerged for the distance value of dtH2 (i.e., distance between the forward line of the underloaded team and the back line of the overloaded team).

By forcing an approach to the underloaded team's back line the NLP participants could have provoked the emergence of critical regions of performance [11] where the balance between the opposing line-forces could be perturbed leading the social neurobiological system to other performance outcomes like the creation of goal scoring opportunities. Critical regions of performance are characterized by low values of interpersonal distances between attackers and defenders that can lead to transitions in system organisational states, with eventual consequences for performance outcomes [11] as previously observed in studies of performance in several other team sports [10], [50]–[52]. In our study, the proximity of the confronting line-forces could also have led to the emergence of critical states, an assumption that needs to be scrutinized in future studies.

On the other hand, the increase in the distance of dtH2 in both groups across SSCGs was attributed to the fact that the underloaded team, because of the numerical disadvantage, prioritized the closing of space near its goal by moving their lines backwards. This result corroborates previous findings regarding behaviors related to the CdtG, and is in accordance with data from previous work by Travassos et al. [49].

The same trend for dtH1 was not so clear in the RLP participants, since the mean distance values of both variables were roughly approximate in the 5v5 and 5v4 treatments, and increasing in the 5v3 treatment. Apparently, the players at that skill level did not press the underloaded team back line when they were in numerical disadvantage. In fact, the opposite occurred in the 5v3 treatment.

With respect to vertical lines, both groups displayed identical values for dtV1 (i.e., the distance between the left line of the overloaded team and the right line of the underloaded team), which increased with numerical differences between teams. On the opposite wing (i.e., dtV2 or the distance between the right line of the overloaded team and the left line of the underloaded team), the space between lines remained relatively constant across SSCGs conditions for both skill groups. This finding signifies that teams maintained relatively the same distances between vertical line-forces across treatments on only one of the two wings of the field. This outcome might have been constrained by other possible factors besides numerical relations, like the players' preferred foot and strategic options.

In sum, the most important effects were registered in the proximity between an overloaded team's attacking line and an underloaded team's defensive line, only in the NLP group. This outcome may have evidenced exploratory performance behaviors of an overloaded team when offensively pressing the reduced number of players in an underloaded team in order to disturb the equilibrium of opposing line-forces to create scoring opportunities. This type of exploratory activity may have emerged in the more skilled group perhaps due to their better technical skills and the capacity to dribble past opponents at critical values of interpersonal distances (see [53] for evidence on critical values of interpersonal distances between attacker-defender dyads in promoting dribbling actions).

## Conclusions

Data from this study shed important insights on co-adaptive behaviors of agents in team sport systems performing under specific task constraints afforded by different numerical relations and skill levels in SSCGs (see Table 3 for a synthesis of main findings). Individual and team coordination tendencies were clearly constrained by the numerical relations between competing teams and the players' skill level. Skill levels provided different action possibilities available to synergistic groups of players, highlighting the importance of adapting training tasks to the players' individual characteristics in order to facilitate the emergence of required team behaviors. Accordingly, the findings of this study support the assumption that teams, here conceptualized as swarming neurobiological superorganisms, possess the ability to co-adapt to performance constraints that can be manipulated by practitioners during practice in SSCGs. Therefore, designing *shared affordances* for specific group tactics to emerge during practice through manipulations of numerical relations seems a feasible pedagogical methodology. In this study we identified the emergent behaviors constrained by different numerical relations in collective systems, which need to be considered when seeking to enhance the acquisition of specific skills and team tactical behaviors during training.

**Table 3:** Synthesis of inter-individual, intra- and inter-team behavioral trends across SSCGs.

Dominant regions	Teams' dispersion	Teams' relative positioning on field	Space between line-forces on wings	Space between line-forces on sectors
Overloaded team players: increased; Underloaded team players: increased;	Overloaded team: maintained; Underloaded team: decreased.	Overloaded team: approached the opponent's goal; Underloaded team: approached own goal.	dtV1: increased with numerical difference; dtV2: maintained across all numerical differences.	dtH1: decreased as the numerical difference increased for NLP; increased on 5v3 for RLP; dtH2: increased with the numerical difference.

Small-sided and conditioned games sequence: (i) 5v5 (ii) 5v4 and (iii) 5v3.

National- and regional-level players displayed the same behavioral trends, except for dtH1 (see table). Differences between groups were observed for the individual dominant regions, means of teams' dispersion and space between dtV2 line-forces.

Future studies should identify the specific *affordances* supporting such tactical behaviors in order to provide deeper understanding of the players' actions and tactical relations during SSCGs. This information is deemed crucial for coaches to regulate their instructions and feedback provided to players.

## References

[1] Duarte R, Araújo D, Correia V, Davids K (2012) Sport teams as superorganisms: implications of biological models for research and practice in team sports performance analysis. Sports Med 42: 633–642. 22715927 10.2165/11632450-000000000-00000

[2] Sumpter DJT (2006) The principles of collective animal behaviour. Philosophical transactions of the Royal Society B: Biological Sciences 361: 5–22. 10.1098/rstb.2005.1733 PMC1626537 16553306

[3] Kesebir S (2012) The Superorganism Account of Human Sociality: How and When Human Groups Are Like Beehives. Pers Soc Psychol Rev 16: 233–261. 22202149 10.1177/1088868311430834

[4] Stearns S (2007) Are we stalled part way through a major evolutionary transition from individual to group? Evolution 61: 2275–2280. 17910743 10.1111/j.1558-5646.2007.00202.x

[5] Richardson MJ, Marsh KL, Isenhower RW, Goodman JRL, Schmidt RC (2007) Rocking together: dynamics of intentional and unintentional interpersonal coordination. Hum Mov Sci 26: 867–891. 17765345 10.1016/j.humov.2007.07.002

[6] Sebanz N, Bekkering H, Knoblich G (2006) Joint action: Bodies and minds moving together. Trends Cogn Sci 10: 70–76. 16406326 10.1016/j.tics.2005.12.009

[7] Schmidt R, Richardson M (2008) Dynamics of interpersonal coordination. In: Fuchs A, Jirsa VK, editors. Coordination: Neural, Behavioral and Social Dynamics. Verlag Berlin Heidelberg: Springer. pp. 281–308.

[8] Marsh KL, Richardson MJ, Baron RM (2006) Contrasting approaches to perceiving and acting with others. Ecol Psychol 18: 1–38.

[9] Hristovski R, Davids K, Araújo D, Passos P (2011) Constraints-induced emergence of functional novelty in complex neurobiological systems: a basis for creativity in sport. Nonlinear Dynamics Psychol Life Sci 15: 175–206. 21382260

[10] Davids K, Button C, Araújo D, Renshaw I, Hristovski R (2006) Movement models from sports provide representative task constraints for studying adaptive behaviour in human movement studies. Adapt Behav 14: 73–94.

[11] Passos P, Araújo D, Davids K (2013) Self-Organization Processes in Field-Invasion Team Sports. Sports Med 43: 1–7. 23315752 10.1007/s40279-012-0001-1

[12] Passos P, Araújo D, Davids K, Gouveia L, Serpa S, et al (2009) Interpersonal pattern dynamics and adaptative behavior in multiagent neurobiological systems: conceptual model and data. J Mot Behav 41: 445–459. 19482724 10.3200/35-08-061

[13] Button C, Chow J-Y, Travassos B, Vilar L, Duarte R, et al.. (2012) A nonlinear pedagogy for sports teams as social neurobiological systems: how teams can harness self-organization tendencies. In: Ovens A, Hopper T, Butler J, editors. Complexity thinking in physical education. Oxon: Routledge. pp. 135–150.

[14] Worthington E (1974) Learning and teaching soccer skills. Hollywood: Wilshire Book Company.

[15] Teodorescu L (1977) Théorie et méthodologie des jeux sportifs; réunis Léf, editor: Les éditeurs français réunis.

[16] Vilar L, Araújo D, Davids K, Bar-Yam Y (2013) Science of winning soccer: emergent pattern-forming dynamics in Association Football. Journal of Systems Science and Complexity 26: 73–84.

[17] Silva P, Garganta J, Araújo D, Davids K, Aguiar P (2013) Shared knowledge or shared affordances? Insights from an ecological dynamics approach to team coordination in sports. Sports Med 43: 765–772. 23794235 10.1007/s40279-013-0070-9

[18] Passos P, Milho J, Fonseca S, Borges J, Araujo D, et al (2011) Interpersonal Distance Regulates Functional Grouping Tendencies of Agents in Team Sports. J Mot Behav 43: 155–163. 21400329 10.1080/00222895.2011.552078

[19] Travassos B, Araújo D, Vilar L, McGarry T (2011) Interpersonal coordination and ball dynamics in futsal (indoor football). Hum Mov Sci 30: 1245–1259. 21683464 10.1016/j.humov.2011.04.003

[20] Withagen R, Poel HJd, Araújo D, Pepping G-J (2012) Affordances can invite behaviour: Reconsidering the relationship between affordances and agency. New Ideas Psychol 30: 250–258.

[21] Gibson J (1979) The ecological approach to visual perception. Hillsdale, NJ: Lawrence Erlbaum Associates.

[22] Stoffregen T, Gorday K, Sheng Y-Y (1999) Perceiving affordances for another person's actions. J Exp Psychol Hum Percept Perform 25: 120–136. 10069029 10.1037//0096-1523.25.1.120

[23] Mark LS (2007) Perceiving the Actions of Other People. Ecol Psychol 19: 107–136.

[24] Witt JK, Sugovic M, Taylor JET (2012) Action-specific effects in a social context: Others' abilities influence perceived speed. J Exp Psychol Hum Percept Perform 38: 715–725. 22103758 10.1037/a0026261

[25] Witt JK, Sugovic M (2012) Does ease to block a ball affect perceived ball speed? Examination of alternative hypotheses. J Exp Psychol Hum Percept Perform 38: 1202–1214. 22201463 10.1037/a0026512

[26] Riley M, Richardson M, Shockley K, Ramenzoni V (2011) Interpersonal synergies. Front Psychol 2: 1–7. 21716606 10.3389/fpsyg.2011.00038 PMC3110940

[27] Davids K, Araújo D, Correia V, Vilar L (2013) How small-sided and conditioned games enhance acquisition of movement and decision-making skills. Exerc Sport Sci Rev 41: 154–161. 23558693 10.1097/JES.0b013e318292f3ec

[28] Araújo D, Silva P, Davids K (in press) Capturing group tactical behaviors in expert team players. In: Baker J, Farrow D, editors. Rotledge Handbook of Sport Expertise: Routledge.

[29] Chow JY, Davids K, hristovski R, Araújo D, Passos P (2011) Nonlinear pedagogy: learning design for self-organizing neurobiological systems. New Ideas Psychol 29: 189–200.

[30] Silva P, Aguiar P, Duarte R, Davids K, Araújo D, et al.. (in press) Effects of pitch size and skill level on tactical behaviours of Association Football players during small-sided and conditioned games. International Journal of Sports Science & Coaching. 10.1080/02640414.2014.961950 25356995

[31] Eccles D (2010) The coordination of labour in sports teams. Int Rev Sport Exerc Psychol 3: 154–170.

[32] Yue Z, Broich H, Seifriz F, Mester J (2008) Mathematical analysis of a soccer game. Part I: individual and collective behaviours. Studies in Applied Mathematics 121: 223–243.

[33] Fonseca S, Milho J, Travassos B, Araújo D (2012) Spatial dynamics of team sports exposed by Voronoi diagrams. Hum Mov Sci 31: 1652–1659. 22770973 10.1016/j.humov.2012.04.006

[34] Frencken W, Lemmink K, Delleman N, Visscher C (2011) Oscillations of centroid position and surface area of soccer teams in small-sided games. Eur J Sport Sci 11: 215–223.

[35] Duarte R, Araújo D, Folgado H, Esteves P, Marques P, et al (2013) Capturing complex, non-linear team behaviours during competitive football performance. Journal of Systems Science and Complexity 26: 62–72.

[36] Duarte R, Fernandes O, Folgado H, Araújo D (2013) Single camera analysis in studying pattern forming dynamics of player interactions in team sports. In: Davids K, Araújo D, Balague N, Button C, Passos P, editors. Complex systems in sport: Routledge.

[37] Gréhaigne J-F, Bouthier D, David B (1997) Dynamic-system analysis of opponent relationships in collective actions in soccer. J Sports Sci 15: 137–149. 9258844 10.1080/026404197367416

[38] Grehaigne J-F, Godbout P (1995) Tactical knowledge in team sports from a constructivist and cognitivist perspective. Quest 47: 490–505.

[39] Gréhaigne J-F (1988) Game systems in soccer from the point of view of coverage of space. In: Reilly T, Lees A, Davids K, Murphy WJ, editors. Science and Football. London: E. & FN Spon. pp. 316–321.

[40] Hughes C (1994) The football association coaching book of soccer tactics and skills. Harpenden: Queen Anne Press.

[41] Rampinini E, Impellizzeri FM, Castagna C, Abt G, Chamari K, et al (2007) Factors influencing physiological responses to small-sided soccer games. J Sports Sci 25: 659–666. 17454533 10.1080/02640410600811858

[42] Johnston R, Watsford M, Pine M, Spurrs R, Sporri D (2013) Assessment of 5 Hz and 10 Hz GPS units for measuring athlete movement demands. International Journal of Performance Analysis in Sport 13: 262–274.

[43] Coutts AJ, Duffield R (2010) Validity and reliability of GPS devices for measuring movement demands of team sports. J Sci Med Sport 13: 133–135. 19054711 10.1016/j.jsams.2008.09.015

[44] Sinnott RW (1984) Virtues of the Haversine. Sky and Telescope 68: 159.

[45] Chatfield C (2003) The analysis of time series: an introduction. New York: Chapman & Hall.

[46] Cohen J (1988) Statistical power analysis for the behavioral sciences. Hillsdale, NJ: Lawrence Erlbaum Associates.

[47] Travassos B, Araújo D, Davids K, Vilar L, Esteves P, et al (2012) Informational constraints shape emergent functional behaviours during performance of interceptive actions in team sports. Psychol Sport Exerc 13: 216–223.

[48] Edelman G, Gally J (2001) Degeneracy and complexity in biological systems. Proc Natl Acad Sci U S A 20: 13763–13713-13768. 10.1073/pnas.231499798 PMC61115 11698650

[49] Travassos B, Araújo D, Duarte R, McGarry T (2012) Spatiotemporal coordination behaviors in futsal (indoor football) are guided by informational game constraints. Hum Mov Sci 31: 932–945. 22672740 10.1016/j.humov.2011.10.004

[50] Passos P, Araújo D, Davids K, Gouveia L, Milho J, et al (2008) Information-governing dynamics of attacker-defender interactions in youth Rugby Union. J Sports Sci 26: 1421–1429. 18923958 10.1080/02640410802208986

[51] Passos P, Araujo D, Davids K, Gouveia L, Serpa S, et al (2009) Interpersonal Pattern Dynamics and Adaptive Behavior in Multiagent Neurobiological Systems: Conceptual Model and Data. J Mot Behav 41: 445–459. 19482724 10.3200/35-08-061

[52] Araújo D, Davids K, Bennett S, Button C, Chapman G (2004) Emergence of sport skills under constraints. In: Williams M, Hodges N, editors. Skill Acquisition in Sport Research, Theory and Practice. UK: Routledge. pp. 409–433.

[53] Duarte R, Araújo D, Gazimba V, Fernandes O, Folgado H, et al (2010) The ecological dynamics of 1v1 sub-phases in association football. Open Sports Sci J 3: 16–18.

